# Characteristics of Patients with Refractory Meibomian Gland Dysfunction Unresponsive to Intense Pulsed Light Treatment Using a Vascular Filter

**DOI:** 10.3390/diagnostics16162532

**Published:** 2026-08-11

**Authors:** Yoo Young Jeon, Nahyun Park, Yea Eun Lee, Chung Min Lee, Sanghyu Nam, Changmin Kim, Ryunhee Lee, Ji Yoon Park, Kyu Sang Eah, Ho Seok Chung, Jae Yong Kim, Hun Lee

**Affiliations:** 1 Department of Ophthalmology, Asan Medical Center, University of Ulsan College of Medicine, Seoul 05505, Republic of Korea; cheese_sauce@naver.com (Y.Y.J.); laurenpark66@gmail.com (N.P.); yeaeun812@gmail.com (Y.E.L.); chungminlee1215@gmail.com (C.M.L.); thehyu@gmail.com (S.N.); kcm8821@naver.com (C.K.); fbsgml97@naver.com (R.L.); jiyoon8068@gmail.com (J.Y.P.); kseah0124@gmail.com (K.S.E.); chunghoseok@gmail.com (H.S.C.); jykim2311@amc.seoul.kr (J.Y.K.); 2 Department of Ophthalmology, Chungnam National University Hospital, Daejeon 35015, Republic of Korea; 3 Department of Ophthalmology, Brain Korea 21 Project, University of Ulsan College of Medicine, Seoul 05505, Republic of Korea; 4 Center for Cell Therapy, Asan Medical Center, Seoul 05505, Republic of Korea

**Keywords:** intense pulsed light, matrix metalloproteinase-9, refractory meibomian gland dysfunction

## Abstract

**Objectives**: To investigate the clinical characteristics of patients with meibomian gland dysfunction (MGD) who are refractory to additional intense pulsed light (IPL) treatment. **Materials and Methods**: This retrospective study included 66 eyes of 66 patients with moderate-to-severe MGD who had previously undergone four sessions of IPL treatment with a 590 nm filter at another clinic and, received an additional four sessions of IPL treatment with a vascular filter. Patients’ subjective assessments before and after the additional IPL treatments were scored on a scale from 0 to 4, where 0 = moderate improvement, 1 = mild improvement, 2 = no change, and 3 = aggravated MGD. Patients who scored 0 or 1 were classified as Group 1 (Responsive group), while those who scored 2 or 3 were classified as Group 2 (Refractory group). **Results**: Among the 66 eyes from 66 patients, Group 1 comprised 32 eyes and Group 2 comprised 34 eyes. Patients in the refractory group, who did not respond to additional IPL treatment, exhibited poor ocular staining scores before additional IPL treatment. The responsive group showed better treatment responses with regard to the Oxford and Sjögren’s International Clinical Collaborative Alliance (SICCA) staining scores (all *p* < 0.001), upper and lower lid meibum quality and meibum consistency, and meibomian gland grade (all *p* < 0.001). Changes in the Oxford staining score (*p* = 0.032), SICCA staining score (*p* = 0.003), meibum quality (*p* < 0.001), meibum consistency (*p* < 0.001), and meibomian gland grade (*p* < 0.001), before and after the additional treatment, were significantly greater in Group 1 than in Group 2. **Conclusions**: Additional IPL treatment with a vascular filter may provide further clinical benefits in selected patients with moderate-to-severe MGD who have previously undergone IPL therapy. Poor baseline ocular surface staining was associated with a lower likelihood of treatment response and may help identify patients who are less likely to benefit from additional IPL treatment.

## 1. Introduction

Dry eye disease (DED) is classified into two main subtypes: aqueous-deficient dry eye and evaporative dry eye, both of which involve pathology affecting the meibomian glands, lacrimal glands, eyelids, tear film, and ocular surface cells [1,2]. The most common cause of evaporative dry eye is meibomian gland dysfunction (MGD) [3]. MGD is characterized by significant changes in the consistency and quantity of meibum secretion, leading to eyelid inflammation and subsequent ocular surface dysfunction. Insufficient lipid secretion from the meibomian glands compromises tear film stability, resulting in dry eye symptoms, despite normal tear secretion [4]. While most cases of MGD are idiopathic, some may be secondary to dermatological conditions such as acne rosacea and Demodex infestation [5].

Various therapies have been used to treat MGD, including warm compresses, lid hygiene practices, dietary supplementation with omega-3 fatty acids, forced meibum expression, topical steroids, topical and oral antibiotics, topical cyclosporine, preservative-free artificial tears, lipid-containing eyedrops [6], topical diquafosol [7], and automated thermal pulsation [8]. Intense pulsed light (IPL) treatment has also been used to improve meibum secretion and quality, stabilize the tear film, and reduce eyelid inflammation [9,10,11]. In 2002, Dr. Toyos was the first to describe the positive effects of IPL on eyelids, noting that patients treated for facial rosacea experienced an improvement in the signs and symptoms of MGD following IPL treatment [12]. Although the exact mechanisms underlying IPL’s beneficial effects are complex and not fully understood, IPL is thought to reduce telangiectasia, eradicate Demodex mites, liquefy meibum through thermal effects, modulate the secretion of anti-inflammatory cytokines, and suppress matrix metalloproteinases [9,13,14]. Several studies have since reported the safety of IPL, with no adverse effects when proper eye protection is used. The use of IPL for treating MGD and dry eye has become increasingly common among eye care providers [14,15,16,17].

Early IPL protocols used for MGD treatment often used a 590 nm filter, but as the usefulness of various filters has been confirmed including acne filters and vascular filters, the types of protocols have become more diversified [18,19,20,21]. A vascular filter is a notched filter that can emit light of shorter wavelengths (530–650 nm) to target the porphyrins and hemoglobin in superficial blood vessels, and light of longer wavelengths (900–1200 nm) to target the hemoglobin in deep blood vessels [18]. It is known to minimize the amount of energy absorbed by melanin and maximize the energy absorbed by oxyhemoglobin. Light wavelengths between 600 and 800 nm, which are mainly absorbed by melanin, can cause pain. The vascular filter blocks light emission in the 650–900 nm wavelength range, minimizing the amounts of light absorbed by melanin pigment while maximizing absorption by hemoglobin [18,21].

Despite the range of available treatments, some patients with MGD are refractory to treatment and do not achieve long-term symptom relief [22]. Wan et al. suggested that meibomian gland dropout may cause refractory MGD and analyzed potential factors affecting long-term meibomian gland recovery. According to their study, age, changes in corneal fluorescein staining, and the meibomian gland area ratio at baseline were potential predictors of meibomian gland recovery [23]. However, there are still patients who do not respond to IPL treatment. Patients in this study were those who had previously received IPL treatment at other clinics and had not improved even with various treatments, including steroid and cyclosporine eyedrops, warm compresses, and eyelid scrubs. Thus, this study assumed that the impact of these treatments on the pre-additional IPL status of the refractory MGD group had been minimal. This study focused on investigating the effect of additional IPL treatment using a vascular filter in patients previously treated with IPL and other treatments for moderate and severe MGD and demonstrating clinical characteristics of patients who are refractory to additional IPL treatment.

## 2. Materials and Methods

A total of 66 eyes from 66 patients were retrospectively enrolled in this study. Among both eyes, the eye at a more severe MGD stage was selected. If both eyes showed the same MGD stage, one of the eyes was randomly selected. Patients with moderate-to-severe MGD who had previously undergone four IPL sessions using a 590 nm filter at another clinic received an additional four IPL treatments at the Department of Ophthalmology at Asan Medical Center. This study was reviewed and approved by the Institutional Review Board of the Asan Medical Center and University of Ulsan College of Medicine (IRB No. 2024-0595) and conducted in accordance with the tenets of the Declaration of Helsinki.

Moderate-to-severe MGD was diagnosed based on the definition established by the international workshop on MGD, which is characterized by moderately or severely altered meibum expressiveness and secretion quality, along with moderate or greater peripheral and central corneal staining [24]. Individuals over 18 years of age with moderate-to-severe MGD, along with subjective symptoms even after four IPL sessions using a 590-nm filter at other clinics, were included [25]. All enrolled patients had Fitzpatrick skin types I–IV. Additional IPL treatment at our clinic was performed in symptomatic patients diagnosed with moderate-to-severe MGD, with a meibum expressibility grade of 2 or higher and lid margin abnormalities such as telangiectasia, plugging, or irregularity.

At baseline, patients underwent clinical evaluation to confirm moderate-to-severe symptomatic MGD and eligibility for additional IPL treatment. The 0–3 subjective response scale was not administered at baseline. One month after receiving four additional IPL treatments with a vascular filter, patients provided a single subjective assessment of symptom change compared with their pretreatment status, scored on a scale from 0 to 3: 0 = moderate improvement, 1 = mild improvement, 2 = no change, and 3 = worsening. Thus, treatment response was not calculated as the difference between subjective scores measured at two time points. Refractoriness after additional IPL treatment was defined as no improvement or even worsening of the subjective symptoms of patients following IPL treatment at our clinic. Patients scoring 0 or 1 were classified as Group 1 (responsive to additional IPL treatment), and those scoring 2 or 3 were classified as Group 2 (refractory to additional IPL treatment). Treatment response was determined using a study-specific, non-validated subjective assessment rather than a standardized symptom questionnaire. Based on these criteria, a subgroup analysis was performed to identify the signs and symptoms of refractory MGD.

Exclusion criteria included a diagnosis of Sjögren’s syndrome, a history of intraocular or ocular surgery within the past six months, glaucoma treated with topical medications, eyelid malposition, ocular infection, non-dry eye ocular inflammation, ocular allergy, other autoimmune diseases, contact lens use during the study period, and pregnancy or lactation.

All patients underwent four additional IPL sessions with a vascular filter at two-week intervals, followed by a one-month post-treatment evaluation. Ophthalmic tests, conducted before and after the additional IPL treatments, included visual acuity, intraocular pressure, tear break-up time (TBUT) using fluorescein, corneal and conjunctival staining scores (Sjögren’s International Clinical Collaborative Alliance [SICCA] staining score and Oxford staining score), Schirmer’s test I without topical anesthesia, and measurement of metalloproteinase-9 (MMP-9) levels. Slit-lamp microscopy was used to evaluate the lid margin (including lid wiper epitheliopathy and vascularity) and meibomian glands. Visual acuity was measured in decimal format, and a single ophthalmologist assessed the TBUT and corneal and conjunctival staining scores via slit-lamp examination.

Meibum quality, consistency, and telangiectasia of the eyelids were evaluated using methods previously described [18,19]. Lid margin telangiectasia was assessed via slit-lamp microscopy and scored from 0 to 3 as follows: 0 = no or slight redness in the lid margin conjunctiva with no telangiectasia crossing the meibomian gland orifices; 1 = redness in the lid margin conjunctiva without telangiectasia crossing the meibomian gland orifices; 2 = redness in the lid margin conjunctiva with telangiectasia crossing the meibomian gland orifices over less than half of the lid’s length; and 3 = redness in the lid margin conjunctiva with telangiectasia crossing the meibomian gland orifices over half or more of the lid’s length. Meibum grades were assessed based on the appearance of meibum when digital pressure was applied to the upper tarsus: grade 0 = clear meibum easily expressed; grade 1 = cloudy meibum expressed with mild pressure; grade 2 = cloudy meibum expressed with more than moderate pressure; and grade 3 = no meibum expression, even with firm pressure. To evaluate meibum consistency, both the upper and lower eyelids were evaluated using meibomian gland expressor forceps. Meibum consistency was graded on a scale from 1 to 3: 1 = oily; 2 = creamy; and 3 = toothpaste-like [26].

MMP-9 levels were measured using the InflammaDry immunoassay (Rapid Pathogen Screening, Inc., Sarasota, FL, USA). The presence of both a blue line in the control zone and a red line in the result zone indicated a positive test result (MMP-9 ≥ 40 ng/mL). The signal intensity of the red line, indicating higher MMP-9 concentration, was graded on a scale from 0 to 4: 0 = none; 1 = trace; 2 = weak positive; 3 = positive; and 4 = strong positive, as described in a previous study [20]. All InflammaDry tests were performed and graded by a single trained examiner using a standardized protocol. Test-line intensity was assessed by direct observation 10 min after testing.

A single trained physician performed IPL treatment on all patients enrolled in the study, and the protocol was identical. For IPL treatment, an M22 Optima IPL device (Lumenis, Yokneam, Israel) with a specific pulse setting (fluence delivering in a 6-5-4 J/cm^2^ decrement pattern) was used [18]. First, ultrasound gel is applied to the skin around the eyes and up to the cheekbones. For the lower eyelid treatment, topical anesthesia was applied first, while both eyelids were closed with protection. Patients received twelve light pulses (with slightly overlapping areas of application) from the left, preauricular area, to the right, preauricular area two times (total: 24 light pulses). For the upper eyelid treatment, a Jaeger lid plate (Katena Products, Denville, NJ, USA) was inserted gently into the space between the bulbar conjunctiva and the upper eyelid. Patients then received two to three light pulses after tenting the lid plate upward to protect the ocular surface. Gentle meibomian gland expression was performed on all four eyelids using Meibomian Gland Compressor (Katena Products, Denville, NJ, USA) immediately after IPL treatment.

Throughout the follow-up period, patients were advised to use loteprednol 0.5% (Lotemax; Bausch & Lomb, Tampa, FL, USA) four times daily, cyclosporine 0.1% (Ikervis; cationic emulsion at 0.1% CsA; Santen Pharmaceutical Co., Ltd., Osaka, Japan) once daily, carbomer gel (Liposic; 2 mg/g carbomer eye gel; Bausch + Lomb), and 0.15% sodium hyaluronate (New Hyaluni; Taejoon, Seoul, Republic of Korea). Additionally, patients were instructed to use warm compresses and perform lid scrubs.

### Statistical Analysis

All statistical analyses were performed using SPSS statistical software for Windows (version 25.0; SPSS Inc., Chicago, IL, USA). The Shapiro–Wilk test was employed to analyze the normality of the data. The Mann–Whitney U test was used to compare clinical parameters between Group 1 (responsive to additional IPL treatment) and Group 2 (non-responsive to additional IPL treatment). A *p*-value of < 0.05 was considered statistically significant.

## 3. Results

Among the 66 eyes from 66 patients, Group 1 comprised 32 eyes and Group 2 comprised 34 eyes. The mean age of Group 1 was 66.2 ± 10.8 years, while the mean age of Group 2 was 65.1 ± 9.1 years. The baseline clinical parameters for both groups are presented in Table 1. No significant differences were observed between the two groups at baseline, except for the Oxford staining score. The Oxford staining scores were 2.5 ± 1.1 in Group 1 and 3.3 ± 1.4 in Group 2, with the responsive MGD group showing significantly lower staining scores (*p* = 0.020).

**Figure 1:**
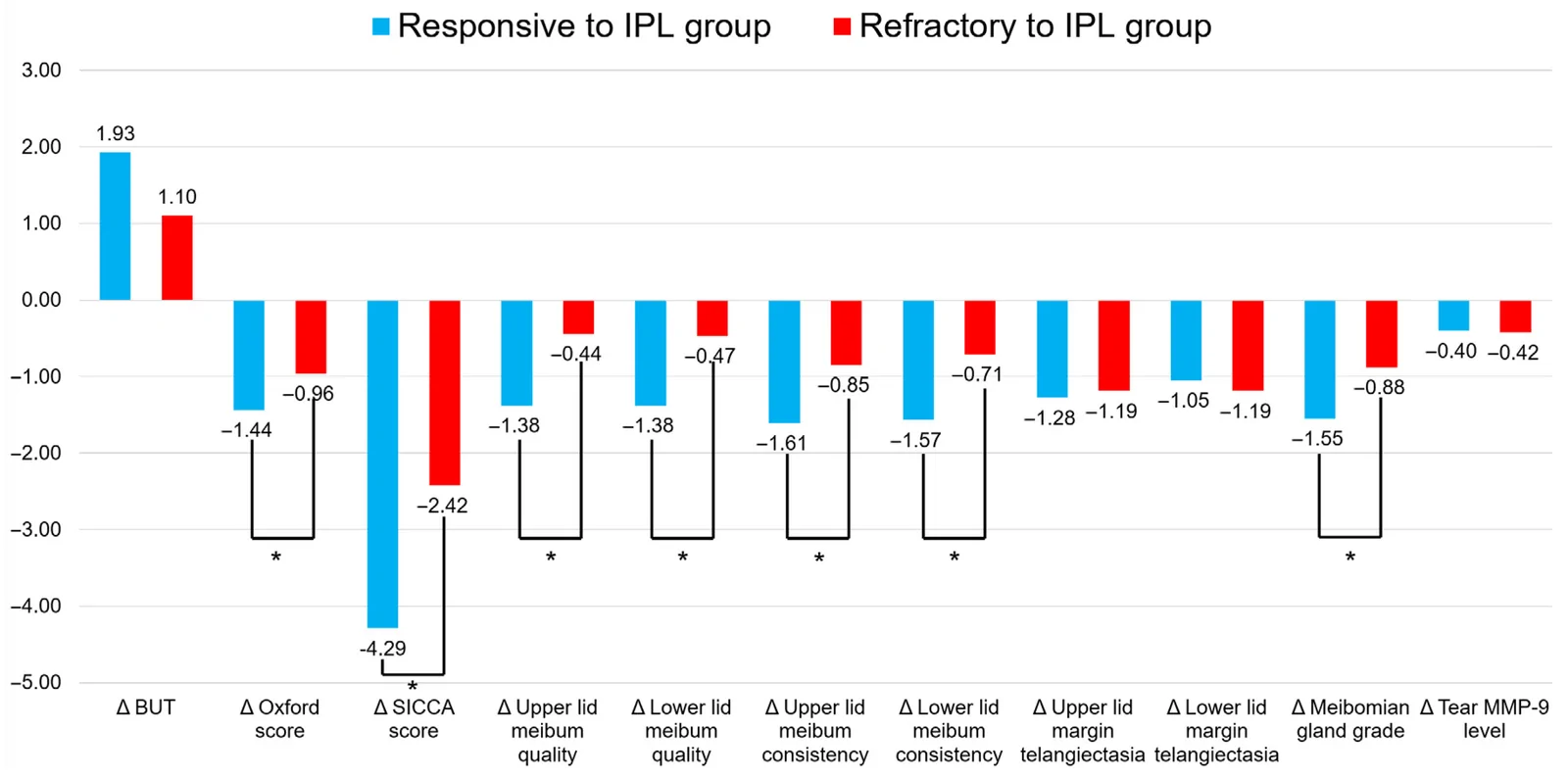
Changes in MGD-related clinical parameters before and after the additional IPL treatment between the responsive and refractory groups. IPL = intense pulsed light; BUT = break-up time; SICCA Sjögren’s International Clinical Collaborative Alliance; MMP-9 = metalloproteinase-9. * *p* < 0.05.

One-month after IPL treatment, the mean TBUT was 3.3 ± 1.1 s in Group 1 and 3.0 ± 1.2 s in Group 2, with no significant difference between the two groups (*p* = 0.199). However, significant differences were observed in the Oxford staining scores (Group 1 = 1.1 ± 0.3 vs. Group 2 = 2.1 ± 1.1, *p* < 0.001) and SICCA staining scores (Group 1 = 2.1 ± 1.9 vs. Group 2 = 4.9 ± 2.4, *p* < 0.001). MMP-9 levels were 1.8 ± 1.4 in Group 1 and 2.2 ± 1.0 in Group 2 (*p* = 0.202) (Table 2). Changes in the Oxford staining score (*p* = 0.032), SICCA staining score (*p* = 0.003), meibum quality (*p* < 0.001), consistency (*p* < 0.001), and meibomian gland grade (*p* < 0.001) before and after treatment were significantly greater in Group 1 than in Group 2 (Figure 1).

## 4. Discussion

This study investigated the clinical characteristics of patients with MGD who are refractory to additional IPL. Improvements in Oxford staining scores, SICCA staining scores, meibum quality, consistency, and meibomian gland grade were significantly greater in the responsive group. Poor baseline ocular surface staining was associated with a lower likelihood of treatment response.

Various treatment methods for MGD have been introduced in numerous studies, each demonstrating varying degrees of effectiveness. Lam et al. reported that self-applied eyelid warming, thermal pulsation, IPL, meibomian gland probing, antibiotics, Manuka honey, and lipid-containing or perfluorohexyloctane eye drops exhibited significant clinical efficacy [5]. Notably, IPL treatment has been highlighted as a highly effective option [5,16]. Despite identical IPL treatment settings, responses varied among patients, prompting this study to investigate the characteristics of those with refractory MGD who did not respond well to additional IPL treatments. Previous studies have identified patient populations that respond well to IPL treatment. Tang et al. reported significant improvements in various parameters, including the Ocular Surface Disease Index (OSDI), TBUT, corneal fluorescein staining score, meibomian glands yielding secretion score, meibomian glands yielding clear secretion, and meibomian glands yielding liquid secretion after three cycles of IPL/meibomian gland expression (MGX) treatments [27]. Characteristics associated with better outcomes after IPL treatment included younger age (*p* = 0.012), longer TBUT (*p* = 0.010), better meiboscore (*p* = 0.012), and less meibomian gland loss (*p* = 0.008) before IPL/MGX [27]. Similarly, in a study by Chen et al. involving 48 patients, univariate analysis identified factors affecting IPL effectiveness. Patients aged 18–39 years, with moderate MGD, higher baseline Schirmer test results, and higher baseline OSDI scores, exhibited a higher effective rate for IPL treatment. MGD severity was strongly associated with IPL effectiveness; patients with moderate MGD had an odds ratio of 5.493 compared to those with severe MGD (*p* = 0.003) [28].

Monitoring the therapeutic effects of IPL is performed using TBUT, the corneal staining score, lid margin telangiectasia, meibomian gland secretion score, and tear MMP-9 expression. Previous studies have demonstrated that MMP-9 can serve as a predictor for the effectiveness and treatment response of IPL [29]. The MMP-9 test is a convenient method for objectively evaluating ocular surface inflammation during outpatient visits. Elevated MMP-9 levels have also been detected in DED patients. Chotikanovich et al. measured tear MMP-9 activity in 46 patients newly diagnosed with DED, and 19 asymptomatic controls [30]. They found that DED patients had a significantly increased MMP-9 activity of 35.57 ± 17.04 ng/mL (*p* < 0.008) compared to controls, whose levels averaged 8.39 ± 4.70 ng/mL. Normal MMP-9 levels in human tears ranged from 3.0 to 41.0 ng/mL, with 90% of individuals having levels below 30 ng/mL. Patients in the highest severity category exhibited a mean MMP-9 activity of 381.24 ± 42.83 ng/mL (*p* < 0.001), which was significantly elevated compared to controls and all lower severity groups [30,31]. Previous studies have confirmed that MMP-9 levels and positivity decreased after IPL treatment compared to baseline [15,19]. Higher tear MMP-9 levels before IPL treatment might be a factor associated with refractoriness for patients with MGD who did not respond to the treatment [19]. Patients were advised to use loteprednol 0.5% and cyclosporine 0.1% throughout the IPL treatment follow-up period, along with warm compresses and lid scrubs. It is well-established that anti-inflammatory corticosteroids, tetracycline derivatives [32,33], and cyclosporine [34] can effectively reduce tear fluid MMP-9 levels. Therefore, since patients with poor responses to IPL treatment exhibited high MMP-9 levels despite the application of anti-inflammatory eye drops, combining various treatment modalities—including more potent anti-inflammatory agents that can lower tear MMP-9 levels—may be beneficial for the treatment of MGD by reducing ocular surface inflammation.

IPL treatment with a vascular filter has special effects, not only because of the specific filter but also because of a system called advanced optimal pulse technology (AOPT; personalized multi-sequential pulsing) [18]. This technology separates every particle of light emitted from the IPL into three pulses, and each pulse can be modulated into a specific strength and duration. When vascular filter-assisted IPL meets the AOPT, there are three resulting advantages. First, HbO2 is converted to Hb, which is then converted to MetHb during the first sub-pulse. Second, denaturation of the targeted tissue occurs during the intervals between each pulse, allowing it to absorb 3–4 times more energy. Third, because of this denaturation, the maximal thermal effect can be obtained with less pulse power and minimal tissue damage. Pulsed light over 900 nm reaches the deeper vasculature for complete thrombosis to deliver thermal energy to MetHb, with a maximal extinction coefficient in 900–1200 nm wavelengths. Therefore, IPL treatment using a vascular filter can allow the energy to be focused on the vascular structure [18,19,20].

This study has some limitations, including a small sample size, a retrospective design, and selection bias. As this is a retrospective study, the sample size was not adequately considered when analyzing medical records [35]. Our findings represent short-term treatment responses rather than long-term treatment success since outcomes were assessed only one month after completion of additional IPL treatment. Importantly, the way the study group was constructed may also have introduced selection bias, as group allocation was based on patient-reported improvements after additional IPL treatment. Patients who were classified as refractory should not necessarily be regarded as true treatment failures. Some patients demonstrated objective improvement without corresponding symptomatic benefit, suggesting symptom–sign discrepancy in MGD. Furthermore, an important limitation of this study is the lack of detailed information regarding previous IPL treatments performed at other clinics, including treatment intervals, devices, settings, and initial clinical responses. Previous IPL treatment may not have followed a standardized protocol, and heterogeneity in treatment intensity and response may have influenced both baseline disease severity and responsiveness to additional IPL treatment. This study did not have access to pre- or post-IPL treatment data from previous IPL sessions at other clinics. Therefore, it is possible that patients who had achieved significant improvements in MGD signs from previous IPL treatments were included in the study. In addition, the lack of a meibomian gland atrophy assessment may also be a limitation of this study. Future studies with larger patient populations and objective meibography-based criteria for defining refractory MGD are needed to identify factors associated with the response to IPL treatment.

## 5. Conclusions

Additional IPL treatment with a vascular filter may provide further clinical benefits in selected patients with moderate-to-severe MGD who have previously undergone IPL therapy. Patients with refractory MGD who did not respond to additional IPL treatment were characterized by poor ocular staining scores before treatment. Poor baseline ocular surface staining may help identify patients who are less likely to benefit from additional IPL treatment.

## Figures and Tables

**Table 1:** Baseline demographics and clinical parameters of the responsive group and refractory group before additional IPL treatment.

	Group 1 (Responsive to Additional IPL)		Group 2 (Refractory to Additional IPL)		*p* Value *
Number of eyes/Patients	32/32		34/34		
Sex (Men/Women)	12/20		8/26		0.167
Age (years)	66.2 ± 10.8		65.1 ± 9.1		0.664
TBUT (s)	1.8 ± 1.3		1.6 ± 0.9		0.626
Oxford score	2.5 ± 1.1		3.3 ± 1.4		0.020
SICCA score	6.4 ± 2.1		7.3 ± 2.3		0.092
Meibum quality	UL	LL	UL	LL	0.143 (UL), >0.999 (LL)
	3.0 ± 0.0	3.0 ± 0.0	2.9 ± 0.2	3.0 ± 0.0	
Meibum consistency	UL	LL	UL	LL	0.386 (UL), 0.105 (LL)
	2.8 ± 0.4	2.9 ± 0.4	2.9 ± 0.3	3.0 ± 0.2	
Lid margin telangiectasia	UL	LL	UL	LL	0.079 (UL), 0.188 (LL)
	2.6 ± 0.5	2.7 ± 0.5	2.8 ± 0.4	2.9 ± 0.4	
Meibomian gland grade	2.7 ± 0.5		2.9 ± 0.3		0.056
Tear MMP-9 level	2.2 ± 1.0		2.6 ± 1.2		0.155

Data are presented as mean ± standard deviation. IPL = intense pulsed light; TBUT = tear break-up time; SICCA = Sjögren’s International Clinical Collaborative Alliance; MMP-9 = metalloproteinase-9; UL = upper lid; LL = lower lid. * Statistical analysis was performed with a Mann–Whitney U test.

**Table 2:** Comparison of MGD-related clinical parameters and subjective symptoms between groups at one month after additional IPL treatment.

	Group 1 (Responsive to Additional IPL)		Group 2 (Refractory to Additional IPL)		*p* Value *
TBUT (s)	3.3 ± 1.1		3.0 ± 1.2		0.199
Oxford score	1.1 ± 0.3		2.1 ± 1.1		<0.001
SICCA score	2.1 ± 1.9		4.9 ± 2.4		<0.001
Meibum quality	UL	LL	UL	LL	<0.001 (UL), <0.001 (LL)
	1.6 ± 0.5	1.6 ± 0.5	2.5 ± 0.5	2.5 ± 0.5	
Meibum consistency	UL	LL	UL	LL	<0.001 (UL), <0.001 (LL)
	1.2 ± 0.4	1.3 ± 0.6	2.0 ± 0.4	2.3 ± 0.4	
Lid margin telangiectasia	UL	LL	UL	LL	0.055 (UL), 0.189 (LL)
	1.3 ± 0.5	1.7 ± 0.7	1.6 ± 0.7	1.7 ± 0.7	
Meibomian gland grade	1.1 ± 0.5		2.0 ± 0.5		<0.001
Tear MMP-9 level	1.8 ± 1.4		2.2 ± 1.0		0.202

Data are presented as mean ± standard deviation. MGD = meibomian gland dysfunction; IPL = intense pulsed light; TBUT = tear break-up time; SICCA = Sjögren’s International Clinical Collaborative Alliance; MMP-9 = metalloproteinase-9; UL = upper lid; LL = lower lid. * Statistical analysis was performed with a Mann–Whitney U test.

## Data Availability

The datasets generated and/or analysed during the current study are not publicly available due to patient privacy concerns and restrictions imposed by the Institutional Review Board. Participants did not provide consent for public sharing of their clinical data. However, the data may be available from the corresponding author on reasonable requests and with appropriate ethical approvals.

## References

[1] Bron A.J., de Paiva C.S., Chauhan S.K., Bonini S., Gabison E.E., Jain S., Knop E., Markoulli M., Ogawa Y., Perez V. (2017). Tfos dews ii pathophysiology report. Ocul. Surf..

[2] Craig J.P., Nichols K.K., Akpek E.K., Caffery B., Dua H.S., Joo C.K., Liu Z., Nelson J.D., Nichols J.J., Tsubota K. (2017). Tfos dews ii definition and classification report. Ocul. Surf..

[3] Nichols K.K., Foulks G.N., Bron A.J., Glasgow B.J., Dogru M., Tsubota K., Lemp M.A., Sullivan D.A. (2011). The international workshop on meibomian gland dysfunction: Executive summary. Investig. Ophthalmol. Vis. Sci..

[4] Rabensteiner D.F., Aminfar H., Boldin I., Schwantzer G., Horwath-Winter J. (2018). The prevalence of meibomian gland dysfunction, tear film and ocular surface parameters in an austrian dry eye clinic population. Acta Ophthalmol..

[5] Lam P.Y., Shih K.C., Fong P.Y., Chan T.C.Y., Ng A.L., Jhanji V., Tong L. (2020). A review on evidence-based treatments for meibomian gland dysfunction. Eye Contact Lens.

[6] Geerling G., Tauber J., Baudouin C., Goto E., Matsumoto Y., O’Brien T., Rolando M., Tsubota K., Nichols K.K. (2011). The international workshop on meibomian gland dysfunction: Report of the subcommittee on management and treatment of meibomian gland dysfunction. Investig. Ophthalmol. Vis. Sci..

[7] Arita R., Suehiro J., Haraguchi T., Maeda S., Maeda K., Tokoro H., Amano S. (2013). Topical diquafosol for patients with obstructive meibomian gland dysfunction. Br. J. Ophthalmol..

[8] Greiner J.V. (2012). A single lipiflow^®^ thermal pulsation system treatment improves meibomian gland function and reduces dry eye symptoms for 9 months. Curr. Eye Res..

[9] Dell S.J. (2017). Intense pulsed light for evaporative dry eye disease. Clin. Ophthalmol..

[10] Wei S., Ren X., Wang Y., Chou Y., Li X. (2020). Therapeutic effect of intense pulsed light (ipl) combined with meibomian gland expression (mgx) on meibomian gland dysfunction (mgd). J. Ophthalmol..

[11] Shin K.Y., Lim D.H., Moon C.H., Kim B.J., Chung T.-Y. (2021). Intense pulsed light plus meibomian gland expression versus intense pulsed light alone for meibomian gland dysfunction: A randomized crossover study. PLoS ONE.

[12] Toyos R., McGill W., Briscoe D. (2015). Intense pulsed light treatment for dry eye disease due to meibomian gland dysfunction; a 3-year retrospective study. Photomed. Laser Surg..

[13] Giannaccare G., Taroni L., Senni C., Scorcia V. (2019). Intense pulsed light therapy in the treatment of meibomian gland dysfunction: Current perspectives. Clin. Optom..

[14] Vora G.K., Gupta P.K. (2015). Intense pulsed light therapy for the treatment of evaporative dry eye disease. Curr. Opin. Ophthalmol..

[15] Chung H.S., Han Y.E., Lee H., Kim J.Y., Tchah H. (2023). Intense pulsed light treatment of the upper and lower eyelids in patients with moderate-to-severe meibomian gland dysfunction. Int. Ophthalmol..

[16] Lee H., Jeon Y.Y., Eah K.S., Park N., Lee Y.E., Han J., Lee C.M., Kim C., Chung H.S., Kim J.Y. (2025). A comparative study of intense pulsed light with two different filters in meibomian gland dysfunction: A prospective randomized study. J. Clin. Med..

[17] Wang M.T., Jaitley Z., Lord S.M., Craig J.P. (2015). Comparison of self-applied heat therapy for meibomian gland dysfunction. Optom. Vis. Sci..

[18] Lee Y., Jang J.H., Nam S., Lee K., Kim J., Kim J.Y., Tchah H., Lee H. (2022). Investigation of prognostic factors for intense pulsed light treatment with a vascular filter in patients with moderate or severe meibomian gland dysfunction. J. Clin. Med..

[19] Jang J.H., Lee K., Nam S.H., Kim J., Kim J.Y., Tchah H., Lee H. (2023). Comparison of clinical outcomes between intense pulsed light therapy using two different filters in meibomian gland dysfunction: Prospective randomized study. Sci. Rep..

[20] Han J.Y., Lee Y., Nam S., Moon S.Y., Lee H., Kim J.Y., Tchah H. (2022). Effect of intense pulsed light using acne filter on eyelid margin telangiectasia in moderate-to-severe meibomian gland dysfunction. Lasers Med. Sci..

[21] Min J.S., Jun I., Kim T.I., Arita R., Seo K.Y. (2024). Comparison of intense pulsed light treatments including upper lid or lateral canthus in patients of meibomian gland dysfunction. J. Clin. Med..

[22] Arita R., Fukuoka S., Mizoguchi T., Morishige N. (2020). Multicenter study of intense pulsed light for patients with refractory aqueous-deficient dry eye accompanied by mild meibomian gland dysfunction. J. Clin. Med..

[23] Wan X., Wu Y., Zhai Z., Yang P., Zhou S., Ye H., Lu Y., Zhou F., Zhou X., Hong J. (2024). Factors affecting long-term changes of meibomian gland in mgd patients. Graefe’s Arch. Clin. Exp. Ophthalmol..

[24] Tomlinson A., Bron A.J., Korb D.R., Amano S., Paugh J.R., Pearce E.I., Yee R., Yokoi N., Arita R., Dogru M. (2011). The international workshop on meibomian gland dysfunction: Report of the diagnosis subcommittee. Investig. Ophthalmol. Vis. Sci..

[25] Gupta P.K., Vora G.K., Matossian C., Kim M., Stinnett S. (2016). Outcomes of intense pulsed light therapy for treatment of evaporative dry eye disease. Can. J. Ophthalmol..

[26] Arita R., Mizoguchi T., Fukuoka S., Morishige N. (2018). Multicenter study of intense pulsed light therapy for patients with refractory meibomian gland dysfunction. Cornea.

[27] Tang Y., Liu R., Tu P., Song W., Qiao J., Yan X., Rong B. (2021). A retrospective study of treatment outcomes and prognostic factors of intense pulsed light therapy combined with meibomian gland expression in patients with meibomian gland dysfunction. Eye Contact Lens.

[28] Chen C., Chen D., Chou Y.-y. (2020). Factors influencing the effectiveness of intense pulsed light for meibomian gland dysfunction. Medicine.

[29] Lanza N.L., Valenzuela F., Perez V.L., Galor A. (2016). The matrix metalloproteinase 9 point-of-care test in dry eye. Ocul. Surf..

[30] Chotikavanich S., de Paiva C.S., Chen J.J., Bian F., Farley W.J., Pflugfelder S.C. (2009). Production and activity of matrix metalloproteinase-9 on the ocular surface increase in dysfunctional tear syndrome. Investig. Ophthalmol. Vis. Sci..

[31] Sambursky R., O’Brien T.P. (2011). Mmp-9 and the perioperative management of lasik surgery. Curr. Opin. Ophthalmol..

[32] De Paiva C.S., Corrales R.M., Villarreal A.L., Farley W.J., Li D.-Q., Stern M.E., Pflugfelder S.C. (2006). Corticosteroid and doxycycline suppress mmp-9 and inflammatory cytokine expression, mapk activation in the corneal epithelium in experimental dry eye. Exp. Eye Res..

[33] Mori M., De Lorenzo E., Torre E., Fragai M., Nativi C., Luchinat C., Arcangeli A. (2012). A highly soluble matrix metalloproteinase-9 inhibitor for potential treatment of dry eye syndrome. Basic Clin. Pharmacol. Toxicol..

[34] Foulks G.N. (2006). Topical cyclosporine for treatment of ocular surface disease. Int. Ophthalmol. Clin..

[35] Boateng E.Y., Abaye D. (2019). A review of the logistic regression model with emphasis on medical research. J. Data Anal. Inf. Process..

